# Clinical outcomes of hypofractionated radiotherapy for thyroid-associated ophthalmopathy

**DOI:** 10.1259/bjro.20200013

**Published:** 2020-09-02

**Authors:** Sang Min Lee, Jung Ho Im, Hyun Soo Shin, Helen Lew

**Affiliations:** 1 Department of Ophthalmology, CHA Bundang Medical Center, CHA University School of Medicine, Seongnam, Republic of Korea; 2 Depratment of Radiation Oncology, CHA Bundang Medical Center, CHA University School of Medicine, Seongnam, Republic of Korea

## Abstract

**Objective:**

To evaluate the safety and effectiveness of hypofractionated orbital radiotherapy applied in the treatment of thyroid-associated ophthalmopathy (TAO) patients.

**Methods:**

Between 2014 and 2018, we retrospectively reviewed the cases of 28 patients with TAO. All patients underwent radiotherapy on both retroocular tissues and received an oral steroid. Patients were divided into two treatment groups: 14 patients received conventional fractionated radiotherapy (20 Gy in 10 fractions), and the second group of 14 patients received hypofractionated radiotherapy (20 Gy in five fractions). The clinical activity score (CAS), NOSPECS (No physical signs or symptoms, Only signs, Soft tissue involvement, Proptosis, Extraocular muscle signs, Corneal involvement, and Sight loss) classification, Hess screen test and binocular single vision (BSV) were evaluated to determine the response to treatment before and at 1 month after radiotherapy.

**Results:**

There were no significant differences in any of the variables between the two treatment groups. In both groups, the CAS and NOSPECS score decreased significantly, and the range of extraocular muscle motility in Hess screen test and BSV improved significantly after radiotherapy (*p* < 0.05). There were no significant differences in CAS, NOSPECS score, Hess screen test or BSV between the two groups. No radiation-related, acute severe toxicity was observed.

**Conclusion:**

Hypofractionated radiotherapy for TAO produced a comparable clinical outcome to that of conventional fractionated radiotherapy. Further case accumulation and long-term follow-up are required to determine if late toxicity occurs and to confirm efficacy.

**Advances in knowledge:**

This is the first study to show that the efficacy and risk of adverse events are comparable between hypofractionated radiotherapy and conventional radiotherapy in the treatment of TAO.

## Introduction

Thyroid-associated ophthalmopathy (TAO) is an autoimmune disease and a common manifestation of Graves’ disease.^1^ In patients with moderate-to-severe and active TAO, treatment is required to relieve infiltrative eye changes and avoid further worsening. The major treatment options include corticosteroids, orbital radiotherapy and decompression surgery.^2^ Although there have been debates on whether radiotherapy is actually effective for TAO, radiotherapy has been recommended as an initial treatment for patients with moderate-to-severe TAO. Previous studies have shown that there is a good response to radiotherapy, with symptom improvement and stabilisation rates exceeding 90%.^3–8^ A total dose of 20 Gy in 10 fractions is generally suggested in the literature.^2,3^

Several retrospective studies and randomised controlled trials have compared treatment efficacy and toxicity among different dose fractionation schedules.^5,9–11^ However, these studies were limited by the small number of patients, and the endpoints for therapeutic responses were heterogeneous. Optimal fractionation schedules and recommended total doses remain unclear. To our knowledge, there has been no study that evaluated the efficacy of hypofractionated radiotherapy (fraction doses > 2 Gy) in patients with TAO. The purpose of this retrospective study was to assess the feasibility and efficacy of hypofractionated radiotherapy and the publication of early clinical results. This study was conducted to determine if a 1-week schedule of hypofractionated radiotherapy (20 Gy in five fractions) is as efficacious and safe as the standard 2-week schedule of conventional fractionated radiotherapy in patients with TAO.

## Methods

The records of all patients with TAO who received radiotherapy between September 2014 and March 2019 were analysed retrospectively. All patients were diagnosed with hyperthyroidism in the Department of Endocrinology and with TAO in the Department of Ophthalmology. The exclusion criteria were as follows: (i) patients who needed surgical treatment, such as orbital decompression; (ii) patients with active intraocular inflammation or infections; (iii) patients with uncontrolled diabetes; (iv) patients with prior retinopathy; and (v) patients younger than 30 years old.

**Figure 1.**
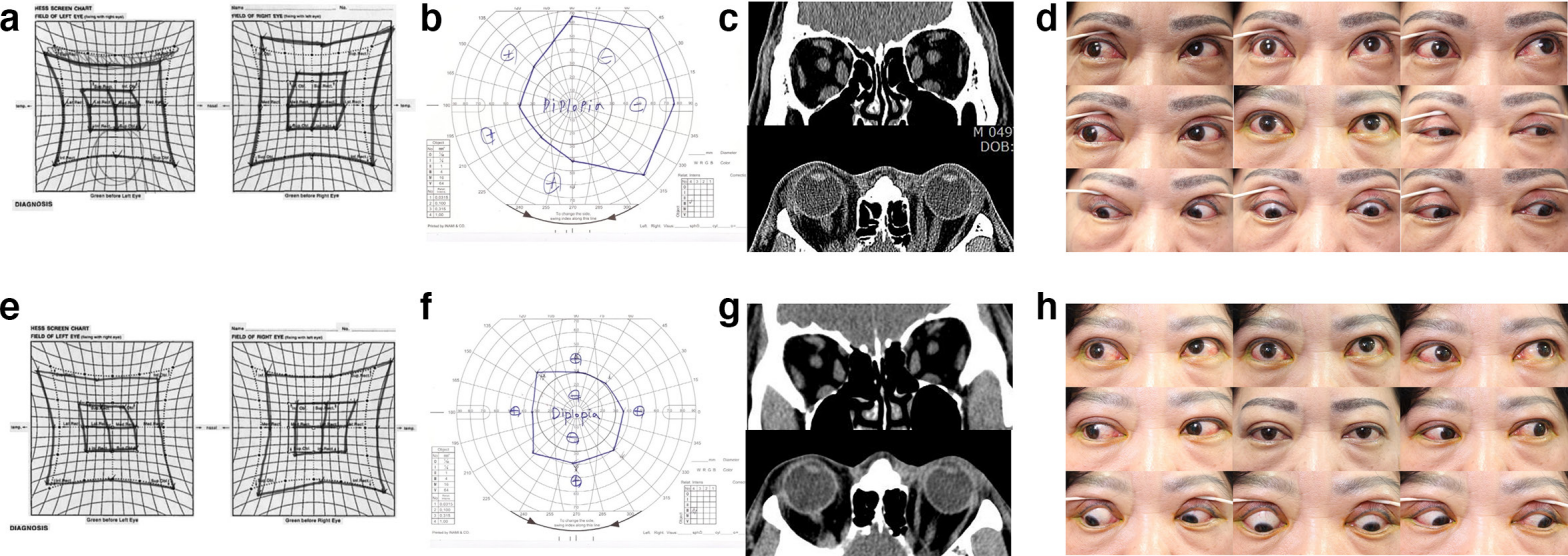
Clinical findings of thyroid ophthalmopathy patients (female, 51 aged) treated with radiotherapy in Group 2. (A, B, C) Hess screen test, binocular single vision (BSV) test, and CT before radiotherapy. (E, F, G) Hess screen test, BSV test, and CT of 2 months after radiotherapy. (D, H) Nine cardinal gaze photographs of patients, before and 2 months after radiotherapy. Extraocular muscle function movement and BSV had improved.

During the patients’ first visit at the clinic, information regarding their smoking history, subjective symptoms, duration of Graves’ disease and TAO were collected. Visual acuity, intraocular pressure, slit lamp examination findings, exophthalmos status with Hertelexophthalmometry, and the presence of diplopia were evaluated at every visit. The severity of symptoms and disease was evaluated using the clinical activity score (CAS),^12^ NOSPECS (No physical signs or symptoms, Only signs, Soft tissue involvement, Proptosis, Extraocular muscle signs, Corneal involvement, and Sight loss) score.^13^ Hypertrophy of orbital fat and extraocular muscles and the associated functions were evaluated with CT, a Hess screen test, and a binocular single vision (BSV) test. In particular, the range of extraocular muscle motility and severity of diplopia were evaluated using the sum of the limitation scale size in four directions on the test chart outside the normal baseline in Hess screen test and the sum of the angle (degree) in eight directions of a non-diplopic region in a BSV test. Representative data of the patients in group II (in group II, 20 Gy was delivered in five fractions), including results of the Hess screen test and BSV test, CT scans, and nine cardinal gaze photographs, are shown in Figure 1(A-H) .

**Figure 2.**
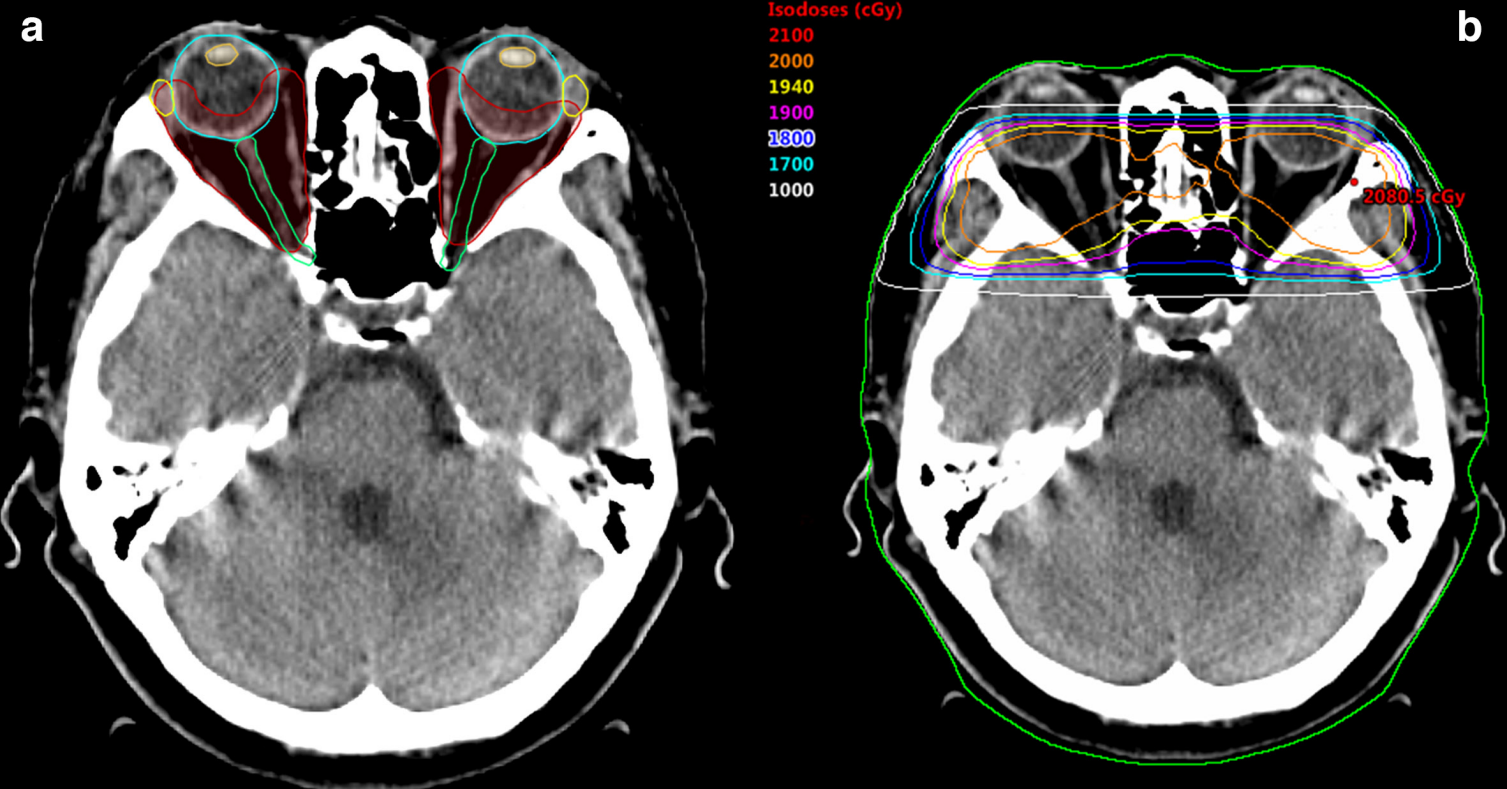
Representative images of a radiotherapy plan for thyroid-associated ophthalmopathy. (A) Target volume delineation (B) Radiation dose distributions.

All patients received three-dimensional conformal radiotherapy using a linear accelerator. CT scans with a slice thickness of 3 mm and without contrast were performed. The clinical target volume encompassing the bilateral retrobulbar tissues, including the extraocular muscle and retrobulbar fat, was delineated (Figure 2A). The treatment isocentre was placed behind the lens to half block the anterior divergence of the beam and minimise the dose to the lens. Opposed lateral 6 MV beams were used with wedges employed to maintain dose homogeneity within the target volume (Figure 2B). In group I, patients received a total dose of 20 Gy in 10 fractions over a 2-week period. In group II, the total dose of 20 Gy was delivered in five fractions of 4 Gy daily for five days in a 1-week period. The following organs at risk were delineated: lenses, eyeballs, optic nerves and lacrimal glands. The normal tissue constraints were as follows: lenses maximum dose <10 Gy, mean dose <3 Gy; eyeballs and optic nerves maximum dose <21 Gy; and lacrimal glands maximum dose <20 Gy. All patients underwent oral steroid therapy. The dose of oral steroids was 40–80 mg per day, administered in proportion to body weight; the dose was reduced by 10 mg per week in subsequent weeks. Three patients who had diabetes underwent oral steroid therapy after controlling their blood sugar levels. Oral steroids were given within 1 month after radiotherapy.

All patients were followed-up at 1-month post-radiotherpay and then every 1–3 months by an endocrinologist and ophthalmologist. One month after radiotherapy, the CAS was assessed and bilateral unilateral examinations were performed, and treatment efficacy and side-effects were evaluated.

Categorical variables were compared using the chi-square test. Continuous variables were compared using the Mann–Whitney test if the data did not have a normal distribution. A non-parametric Wilcoxon’s rank test was performed to assess the statistical significance of the differences between the two groups before and after radiotherapy. A *p*-value of less than 0.05 indicated statistical significance.

## Results

Twenty-eight patients (14 patients per groups) were included in the analysis. The median follow-up periods of all patients, group I, and group II were 19 months (range, 1–59 months), 36 months (range, 4–59 months), and 16 months (range, 1–35 months), respectively. Patient characteristics are shown in Table 1. The chief complaint of most patients was diplopia or exophthalmos. In both groups, 11 patients complained of diplopia, and three patients in group I and two patients in group II complained of exophthalmos. Also, two patients in group I and three patients in group II complained of lid or periocular swelling. Radiotherapy was initiated over a minimum period of 1 month to a maximum of 12 months after the onset of ocular inflammatory signs, with a median period of 1 month. There were no significant differences with respect to the variables considered between groups I and II.

**Table 1.** Comparisons of patient characteristics between treatment groups

	Group I (*n* = 14)	Group II (*n* = 14)	Total (*n* = 28)	*p*
Age (years)	54.9 ± 13.2	59.0 ± 10.4	56.9 ± 11.9	0.511
Sex				1.000
Male	5 (35.7)	5 (35.7)	10 (35.7)	
Female	9 (64.3)	9 (64.3)	18 (34.3)	
Smokers	1 (7.1)	2 (14.3)	3 (10.7)	0.769
Hypertension	6 (42.9)	4 (28.6)	10 (35.7)	0.449
Diabetic mellitus	1 (7.1)	3 (21.4)	4 (24.3)	0.297
Axial length (mm)	23.79 ± 1.02	23.80 ± 1.20	23.79 ± 1.10	0.964
Intraocular pressure (mmHg)	17.0 ± 5.4	17.7 ± 3.6	17.4 ± 4.6	0.582
Duration of hyperthyroidism (years)	1.1 ± 1.4	2.3 ± 3.5	1.7 ± 2.7	0.635
Duration of TAO (months)	1.9 ± 3.0	2.5 ± 3.1	2.2 ± 3.0	0.642
Thyroid hormone				
T3	4 (28.6)	3 (21.4)	7 (25.0)	0.769
Free T4	3 (21.4)	3 (21.4)	6 (21.4)	1.000
Thyroid antibody				
Thyroid stimulating antibody	13 (92.9)	13 (92.9)	26 (92.9)	1.000
TSH receptor antibody	9 (64.3)	8 (57.1)	17 (60.7)	0.769
Anti thyroid peroxidase antibody	3 (21.4)	7 (50.0)	10 (35.7)	0.21
Thyroglobulin antibody	0 (0.0)	2 (14.3)	2 (7.1)	0.541

TAO, thyroid-associated ophthalmopathy; TSH, thyroid stimulating hormone

a Data are presented as the means ± standard deviation or numbers with percentages in parentheses.

The outcome measures that were assessed before and after radiotherapy are summarised in Table 2. For all patients in groups I and II, CAS and NOSPECS scores decreased significantly post-radiotherapy compared with pre-radiotherapy scores (*p* < 0.05), and the range of extraocular muscle motility in Hess screen test and BSV also improved significantly post-radiotherapy (*p* < 0.05). At least one NOSPECS parameter decreased in all 28 patients, and at least one CAS parameter decreased in 27 patients (96.4%). BSV improved in 20 patients (71.4%). There was no significant change in exophthalmos status pre- and post-radiotherapy. However, post-radiotherapy, there were no significant differences between groups I and II with respect to CAS, NOSPECS scores, the range of extraocular muscle motility in Hess screen test, or BSV improvement (*p* = 0.910, *p* = 0.769, *p* = 0.748, and *p* = 0.329, respectively).

**Table 2.** Clinical findings of thyroid-associated ophthalmopathy patients treated with conventional (group I) and hypofractionated (group II) radiotherapy

	Group I (*n* = 14)			Group II (*n* = 14)			*p*a
	Pre-treatment	Post-treatment	*p*b	Pre-treatment	Post-treatment	*p*b	
CAS	3.4 ± 0.6	1.3 ± 0.8	0.001	3.7 ± 0.8	1.4 ± 0.9	0.001	0.910
NOSPECS	9.7 ± 2.5	4.1 ± 1.5	0.001	10.9 ± 2.5	3.9 ± 1.7	0.001	0.769
Hess screen test	3.1 ± 4.3	1.2 ± 1.6	0.001	2.5 ± 6.1	1.0 ± 2.6	0.001	0.748
BSV (^o^)	172.1 ± 172.8	231.4 ± 193.9	0.008	180.7 ± 175.5	298.6 ± 177.8	0.004	0.329
Exophthalmometry (mm)	18.09 ± 2.63	18.05 ± 2.50	0.813	19.05 ± 31.12	18.82 ± 2.58	0.302	0.449

CAS, clinical activity score; BSV, binocular single vision (^o^)

a *p*-values were calculated using the Mann–Whitney test.

b *p*-values were calculated using the Wilcoxon signed-rank test.

One patient in group I and two patients in group II experienced recurrence after radiotherapy. Twenty-five of the 28 patients did not experience recurrence or worsening of TAO after radiotherapy and required no further treatment for TAO during the follow-up period. Radiotherapy was well-tolerated, with no patient experiencing severe acute treatment reaction necessitating a treatment break. In addition, no radiation-related acute toxicity was observed, with the exception of bilateral periocular redness observed in one group II patient. No late toxicity was observed in all patients during the follow-up period.

## Discussion

This retrospective study investigated the safety and effectiveness of hypofractionated radiotherapy in patients with TAO and compared the outcomes between standard fractionated radiotherapy (group I, 20 Gyin 10 fractions) and hypofractionated radiotherapy (group II, 20 Gy in five fractions). Statistically significant decreases in post-radiotherapy CAS and NOSPECS scores were observed in groups I and II. In both groups, the post-radiotherapy Hess screen test and BSV improved significantly compared with the pre-radiotherapy value. No statistically significant differences between groups I and II were observed for CAS, NOSPECS score, the range of extraocular muscle motility in Hess screen test,or BSV. The study results revealed that standard fractionated radiotherapy and hypofractionated radiotherapy are equally effective for patients with TAO.

Based on previous research, combined treatment with radiotherapy and corticosteroids should provide synergetic effects. Previous randomised controlled and retrospective studies showed that the combined treatment was more effective than either radiotherapy or corticosteroids alone.^3,14–18^ Combined treatment reduced the risk of compressive optic neuropathy^17^ and significantly lowered the recurrence rate of inflammation.^14^ Combined treatment can prevent the need for long-term use of corticosteroids and reduce the need for further invasive procedures, including orbital decompression. In this study, all patients were treated with combined radiotherapy and corticosteroids. All patients achieved a level of symptom palliation, and all but three patients experienced no recurrence throughout the follow-up period and did not require any further steroids or orbital decompression. The combination of radiotherapy and glucocorticoids may be an effective treatment for TAO.

The most commonly used radiotherapy dose and fractionation routine are a total dose of 20 Gy applied as 10 fractions of 2 Gy each,^2–4,7,14,15,17,18^ however, the optimal radiotherapy dose has not been sufficiently investigated. A total dose of 30 Gy did not improve outcome with respect to any of the ophthalmic parameters^11^; thus, a higher cumulative dose did not provide further benefits. Based on daily equivalent doses in terms of 2 Gy fractions, five fractions of 4 Gy (group II) resulted in a higher total dose than 10 fractions of 2 Gy (group I).^19^ Accordingly, the current study also found that no further benefits were obtained from a total dose of 20 Gy applied over five fractions compared to 20 Gy applied over 10 fractions. The rationale of using radiotherapy for TAO is to suppress the activated T lymphocytes and fibroblasts in the orbit. As these cells are very sensitive to radiation, lower doses of radiotherapy may be effective in suppressing them. A radiotherapy dose higher than 20 Gy applied over 10 fractions is not recommended. Therefore, it would be suggested to reduce the fraction size to less than 4 Gy (*i.e.,* fraction size of 3 Gy) when conducting five-fraction schedule of hypofractionated radiotherapy upon a TAO patient.

One study showed that the 1 Gy/week radiotherapy protocol (20 divided fractions of 1 Gy weekly over 20 weeks) was effective and tolerated in TAO patients.^10^ The longer treatment duration, however, makes this treatment schedule less practical and inconvenient for patients compared to standard fractionated radiotherapy. Two studies reported that delivering a small fraction of the lower total radiation dose yielded results similar to those obtained with the standard fractionation of 16–20 Gy in 8–10 fractions.^9,10^ One study showed that the total doses of 2.4 and 16 Gy in eight fractions were equally effective,^9^ and another reported that similar response rates were observed when the doses of 10 and 20 Gy in 10 fractions were used.^10^ One study found, however, that the vertical motility significantly improved in the group with 16 and 20 Gy irradiation than in the group with 12 Gy irradiation.^5^ A radiotherapy dose less than 12 Gy may be suboptimal for TAO patients. Thus, the dose of 16–20 Gy in 8–10 fractions is considered the optimal total dose for radiotherapy for TAO.

Conducting radiotherapy during the active phase is recommended because the ocular movement restriction progresses to fibrotic changes, which require surgical management. Once inflammatory infiltration has been replaced by fibrosis, radiotherapy is usually less effective.^6,18^ Patients with active disease who develop signs and symptoms of TAO within 6–7 months before treatment seem to respond better to radiotherapy than those with long-standing TAO in which fibrosis has already occurred.^20,21^ In this study, 26 of the 28 patients (93%) received radiotherapy within 6 months after the onset of ocular inflammatory signs, and all patients responded to the treatment. The results of the previous and current studies indicate that radiotherapy may be effective in recent-onset TAO.

The safety of orbital radiotherapy is well-established. When radiotherapy is properly performed using three-dimensional conformal radiotherapy and the recommended dose, acute side-effects are rare.^4,7,8,18^ This study also found that acute adverse effects related to radiotherapy are minimal. None of the patients in this study suffered from severe long-term complications, including retinopathy or early cataracts, during the follow-up period, which is consistent with the results of other studies.^4,14,15,17,18^ Cases of radiation-related cataracts, however, were reported in studies that evaluated long-term sequelae.^6^ As the follow-up period in this study was too short, however, further research is needed to confirm the safety of hypofractionated radiotherapy for TAO.

Several classification systems have been used for TAO to evaluate the disease severity and treatment efficacy. The CAS and NOSPECS classifications are commonly used in the evaluation of TAO,^2,5–8,10,11,14,16,20^ and some previous studies used the BSV test^14,22^ and Hess screen test^14,23^ to evaluate TAO. No method is both specific and completely reliable, and both the CAS^24^ and NOSPECS classifications^25^ have limitations in the evaluation of TAO. Therefore, in this study, we assessed the severity of the disease and the response using four methods (CAS, NOSPECS score, BSV test, and a Hess screen test) instead of only one or two. As a result, the patient’s symptoms were alleviated in all the four methods. The results of this study are thought to be reliable.

This study had a few limitations. First, it was a non-randomised retrospective study with a probability of unrecognised bias. Second, the study enrolled a small number of patients. Larger studies overcoming these limitations are needed in the future.

The results of this retrospective study suggest that, compared to standard fractionated radiotherapy, hypofractionated radiotherapy using a total dose of 20 Gy in five fractions does not provide significantly different outcomes with respect to efficacy or toxicity. Additional larger scale studies will be required to determine the efficacy of hypofractionated radiotherapy, and long-term follow up is needed to determine whether late-stage toxicity occurs post-treatment.
